# Conversion therapy combined with ALPPS for the treatment of intrahepatic cholangiocarcinoma: a case report

**DOI:** 10.3389/fonc.2025.1542955

**Published:** 2025-03-21

**Authors:** Hengyu Tian, Qinghua He, Chidan Wan

**Affiliations:** ^1^ Department of Hepatobiliary Surgery, Union Hospital, Tongji Medical College, Huazhong University of Science and Technology, Wuhan, China; ^2^ Shenzhen Traditional Chinese Medicine Hospital/The Fourth Clinical Medical College of Guangzhou University of Chinese Medicine, Shenzhen, China

**Keywords:** intrahepatic cholangiocarcinoma, conversion therapy, GEMOX, ALPPS, immunotherapy, targeted therapy

## Abstract

**Rationale:**

Intrahepatic cholangiocarcinoma (ICC) is a highly malignant liver tumor with limited treatment options for advanced cases. Conversion therapy combining immunotherapy, targeted therapy, and chemotherapy offers a promising approach to enable surgical resection, which remains the only curative option.

**Patient concerns:**

A 67-year-old male presented with right upper abdominal pain for two months. Imaging and biopsy confirmed advanced ICC (Stage IV), with a 98 mm tumor, lymphadenopathy, and elevated tumor markers (CA199: 1190.4 U/ml). The disease was deemed unresectable.

**Diagnosis:**

The patient was diagnosed with advanced ICC involving a large hepatic mass, lymph node metastasis, and insufficient liver reserve for conventional resection.

**Interventions:**

The patient received six months of oxaliplatin plus gemcitabine (GEMOX), lenvatinib, and toripalimab, achieving significant tumor regression. A two-step ALPPS procedure was then performed, comprising portal vein ligation and right hepatectomy.

**Outcomes:**

The treatment reduced tumor size (98 mm to 60 mm), normalized tumor markers, and improved liver reserve. Postoperative pathology confirmed >80% tumor remission with negative margins. At 12 months post-surgery, the patient remained disease-free.

**Lessons:**

This case demonstrates that advanced ICC can be downstaged with systemic therapy, enabling resection via ALPPS. The combination of GEMOX, lenvatinib, and toripalimab is an effective and safe conversion therapy regimen. This approach may serve as a model for managing similar advanced cases.

## Introduction

Intrahepatic cholangiocarcinoma (ICC) is the second most common primary malignant liver tumor, accounting for 10%-15% of all primary liver tumors, with a rising incidence rate. Due to its insidious clinical presentation, high aggressiveness, and poor treatment outcomes, ICC is associated with a very high mortality rate (1). Radical surgery remains the most effective treatment for early-stage ICC. However, 60%-88% of patients are diagnosed at an advanced stage, thereby missing the opportunity for surgical treatment (2).

In recent years, with the rapid development of interventional therapies, targeted therapies, and immunotherapies, conversion therapy has enabled some initially unresectable ICC patients to become eligible for surgery, thereby improving their prognosis (3, 4). This case report presents an advanced unresectable ICC patient successfully treated with a regimen of oxaliplatin plus gemcitabine (GEMOX), lenvatinib, and toripalimab, followed by Associating Liver Partition and Portal Vein Ligation for Staged Hepatectomy (ALPPS), to achieve radical tumor resection.

## Case presentation

A 67-year-old male was admitted to the hospital in April 2023 with a two-month history of right upper abdominal pain. Abdominal ultrasound and computed tomography (CT) scans revealed a space-occupying lesion in the liver. The patient’s medical history included hypertension with an unknown highest recorded blood pressure and irregular use of antihypertensive medication. The patient had a long history of alcohol consumption. There was no family history of cancer. Physical examination showed no significant abnormalities.

Laboratory investigations revealed CA199 at 1190.4 U/ml, CA125 at 2858.9 U/ml, CEA at 8.3 ng/ml, AFP at 1.0 ng/ml, AFP-L3% at <0.5%, and PIVKA-II at 28 mAU/ml.

**Figure 1:**
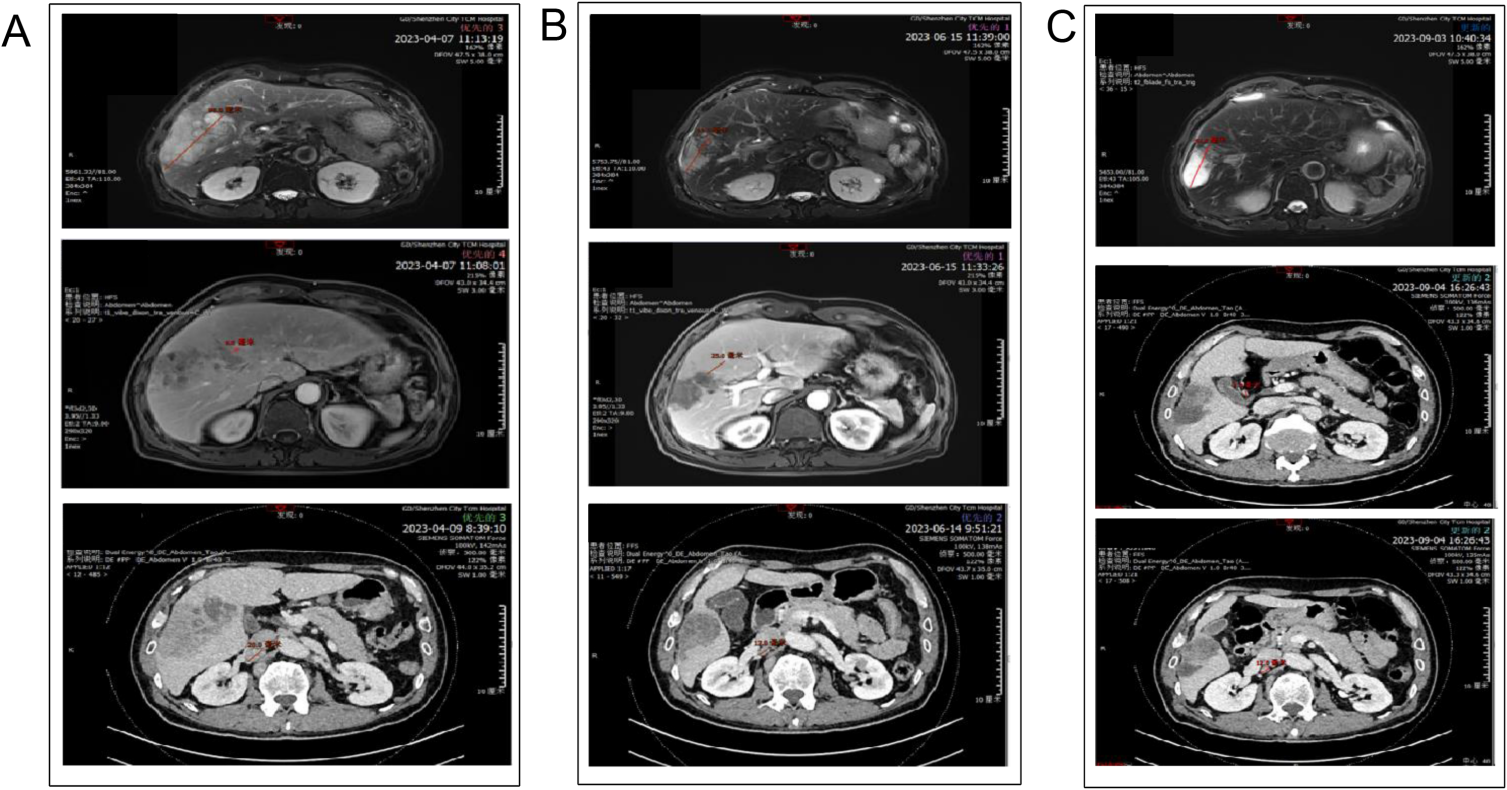
Computed tomography (CT) scans. **(A)** CT scan of the patient in April 2023. **(B)** CT scan of the patient in June 2023. **(C)** CT scan of the patient in September 2023.

Liver-enhanced magnetic resonance imaging (MRI) showed an abnormal signal shadow in the right lobe of the liver measuring 65 mm × 98 mm × 89 mm, consistent with ICC with multiple intrahepatic foci, bile duct invasion, and enlarged retroperitoneal lymph nodes suggestive of metastasis. Abnormal enhancement in the 9th thoracic vertebra raised suspicion of metastatic involvement. Contrast-enhanced CT of the chest and abdomen revealed a large liver mass measuring 55 mm × 96 mm, with local invasion of the right portal vein and multiple enlarged lymph nodes (the largest measuring 20 mm) (**Figure 1A**), also consistent with metastasis. A liver tumor biopsy confirmed ICC based on morphology and immunomarkers. Additionally, the CT and MRI reports described an irregular liver surface. Therefore, we preoperatively suspected that the patient had alcohol-related liver cirrhosis and assessed liver function using the Child-Pugh score. The patient’s condition was classified as Child-Pugh grade A, with a score of 5 points. According to the 8th edition of the AJCC Cancer Staging Manual, the clinical stage was determined as T2N1M1 (Stage IV).

The multidisciplinary team (MDT) at our hospital evaluated the case and determined the tumor was unsuitable for radical resection. The first-line treatment for advanced ICC, GEMCIS (gemcitabine and cisplatin), offers a median overall survival of 11.7 months. GEMOX (gemcitabine and oxaliplatin) is another common regimen, particularly for Asian patients, with a comparable OS. Considering the poor survival outcomes of chemotherapy alone and evidence suggesting the efficacy of molecular targeted drugs and immunotherapy, the MDT recommended a combination of GEMOX, lenvatinib, and toripalimab as conversion therapy to downstage the tumor. After informed consent, the patient began systemic treatment in April 2023. Oxaliplatin was administered intravenously at 150 mg on days 1 and 8 of a three-week cycle. Gemcitabine was given intravenously at 1.8 g on days 1 and 8 of the same cycle. Lenvatinib was prescribed orally at 8 mg per day, and toripalimab was administered intravenously at 240 mg every three weeks. The patient underwent regular monitoring of blood counts, liver and kidney function, and tumor markers before each treatment cycle.

After two cycles, enhanced MRI showed that the maximum tumor diameter had reduced from 98 mm to 60 mm. Enhanced CT revealed no invasion of the right portal vein (**Figure 1B**). Tumor markers also decreased significantly, with CA199 declining from 1190.4 U/ml to 87.5 U/ml and CA125 from 2858.9 U/ml to 173.5 U/ml. According to mRECIST criteria, the tumor response was classified as partial response by June 2023.

**Figure 2:**
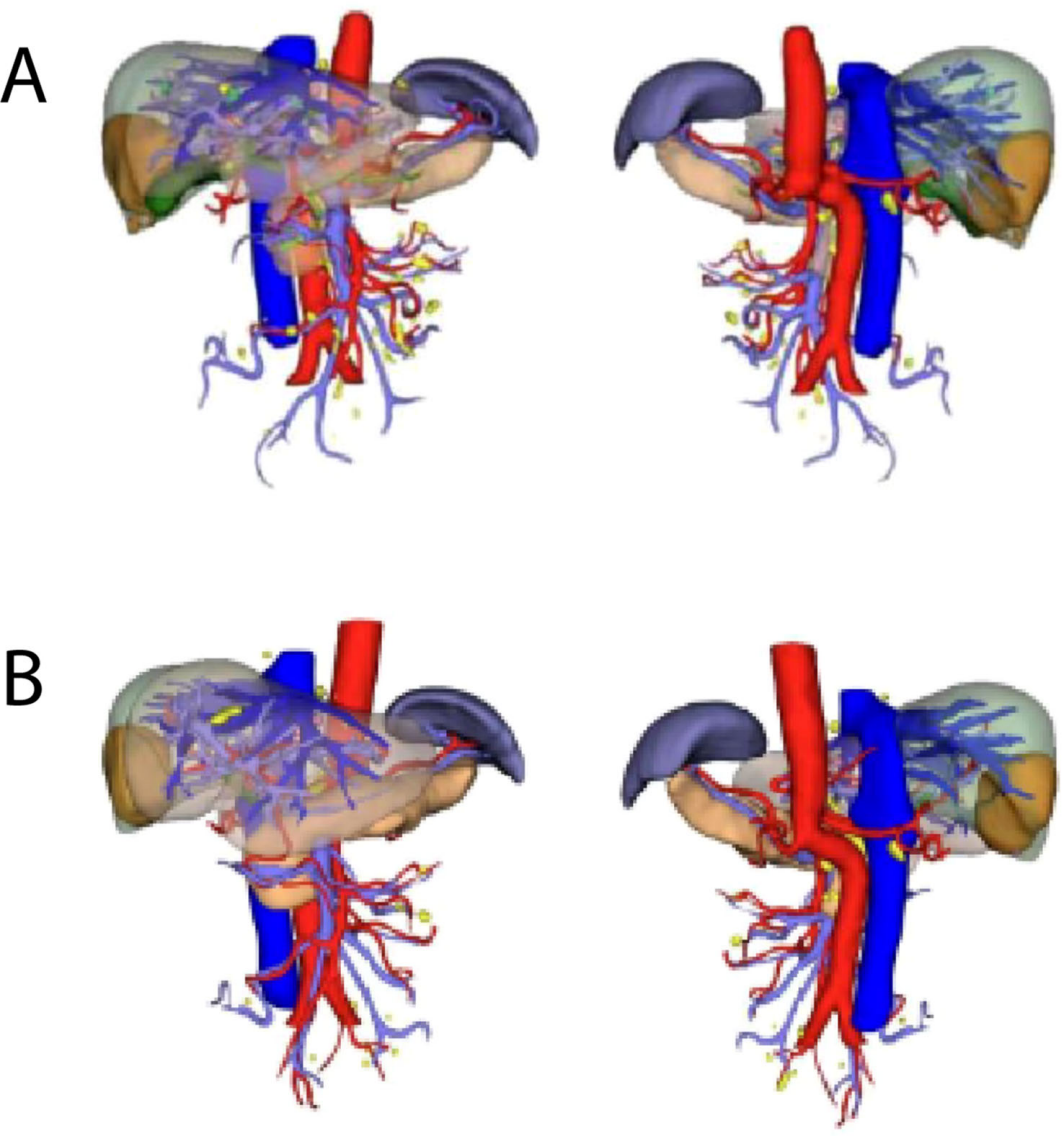
Three-dimensional reconstruction. **(A)** Three-dimensional visualization of the patient’s liver in September 2023. **(B)** Three-dimensional visualization of the patient’s liver in October 2023.

Following three additional treatment cycles, including one session of hepatic artery embolization, enhanced MRI in September 2023 revealed increased tumor necrosis despite stable size. Tumor markers normalized. CT showed a low-density mass in the right lobe (44 mm × 62 mm × 64 mm) with residual metabolic activity (**Figure 1C**). Metabolically active supraclavicular lymph nodes were biopsied and found negative for malignancy. The tumor was assessed as stable disease. Following MDT discussions, the patient was deemed eligible for surgery. Insufficient liver reserve (ICG R15 of 15.8%, residual liver volume of 40.96%) (**Figure 2A**) precluded a traditional right hepatectomy, so ALPPS was chosen to mitigate the risk of postoperative liver failure.

**Figure 3:**
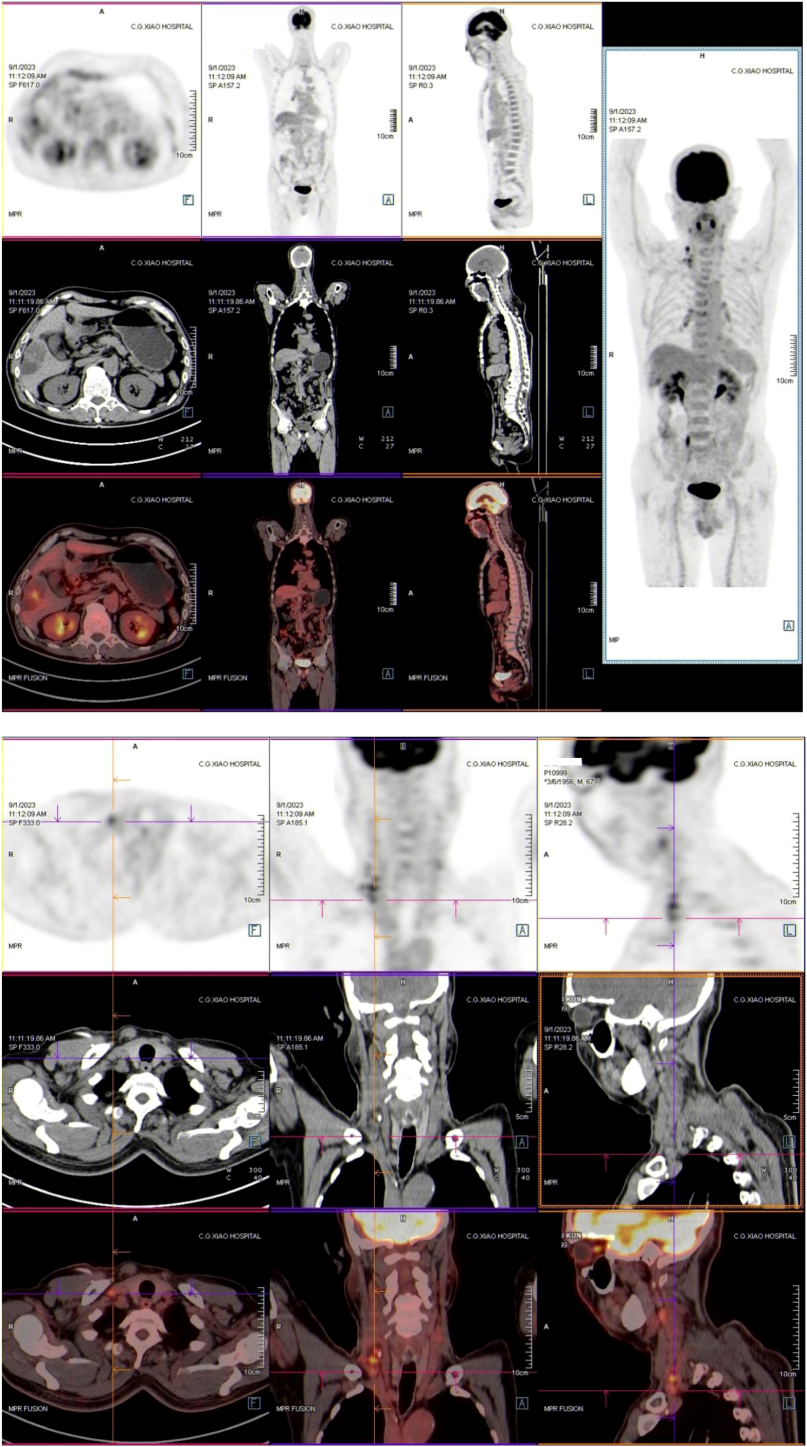
Full body PET-CT images of the patients in September 2023.

**Figure 4:**
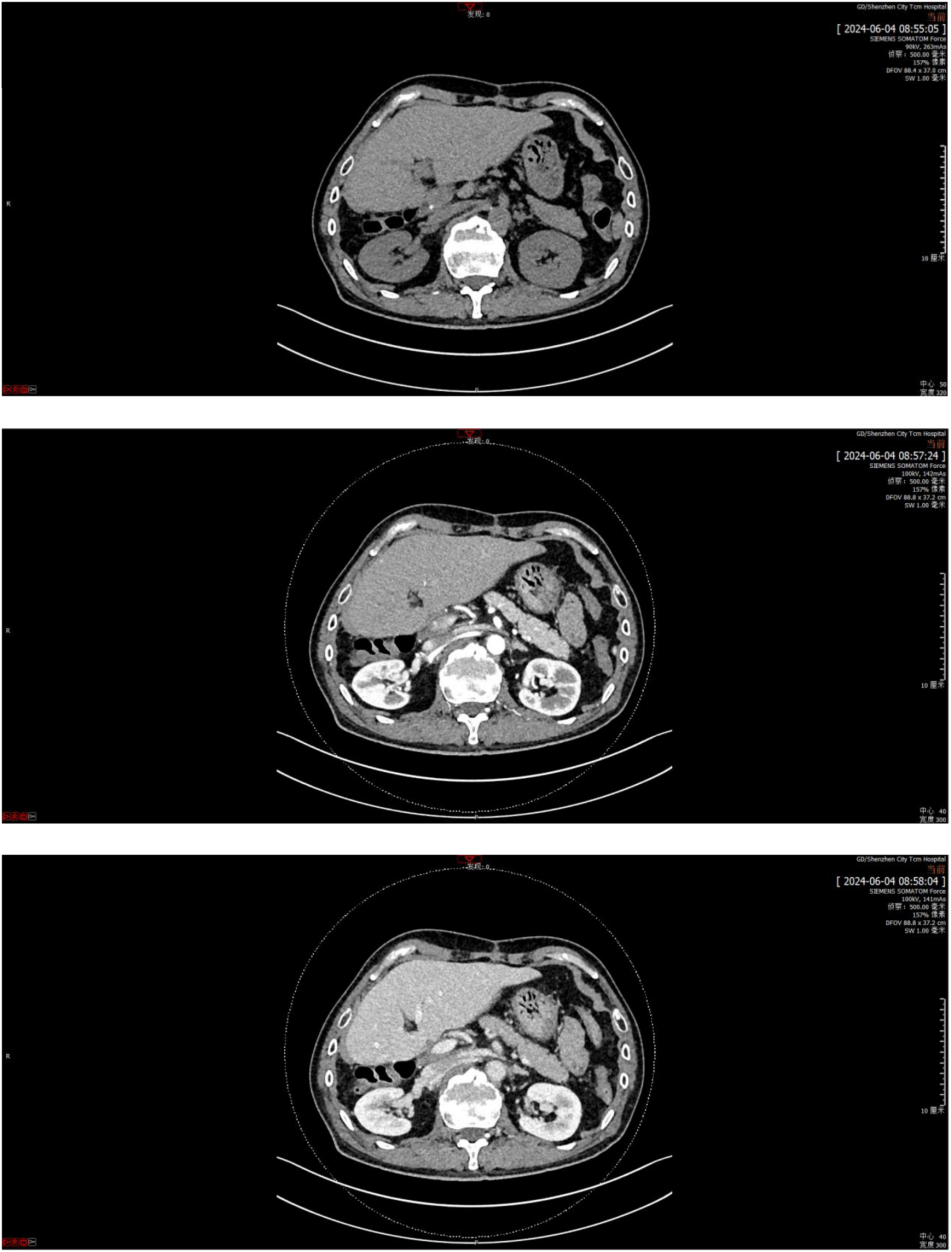
CT scan of the patient at 12 months post-surgery.

The first stage of ALPPS was performed in September 2023, consisting of cholecystectomy, right portal vein ligation, and right liver parenchymal division. Full body PET-CT images of the patient were shown in **Figure 3**. On day 37 post-surgery, reassessment showed improved liver function, with an ICG R15 of 5.8% and residual liver volume of 55.14% (**Figure 2B**). In October 2023, the second stage involved right hepatectomy and lymph node dissection. Pathological analysis confirmed moderately differentiated ICC with a tumor size of 65 mm × 60 mm × 40 mm, >80% tumor necrosis, and negative margins. No metastatic carcinoma was detected in the lymph nodes. The patient recovered well and was discharged. At 12 months post-surgery, no recurrence or distant metastasis was observed. The CT scan images were shown in **Figure 4**.

## Discussion

ICC is a highly malignant biliary tumor with a poor prognosis. Early diagnosis and radical surgical resection are the only options for long-term survival in ICC patients. However, only 20%-30% of patients are eligible for radical resection at the time of diagnosis (2, 5). Advanced unresectable patients often do not survive more than 12 months after diagnosis, even with standard treatment such as GEMCIS, which offers a median overall survival of only 11.7 months (6). Therefore, there is an urgent need to improve the surgical resection rate to enhance the prognosis of ICC patients. The feasibility of surgical resection is mainly determined based on factors such as tumor size, vascular involvement, lymph node metastasis, distant metastasis, and residual liver function. The European Association for the Study of the Liver (EASL) guidelines classify ICC according to the TNM staging system outlined in the 8th edition of the American Joint Committee on Cancer (AJCC) Cancer Staging Manual, designating stage III and IV ICC as unresectable (7). In this case, imaging examinations indicated local invasion of the right hepatic lobe portal vein, involvement of the intrahepatic bile ducts in the right lobe, and the formation of multiple intrahepatic metastatic lesions, all of which suggest a poor prognosis. Additionally, the imaging report mentioned multiple enlarged retroperitoneal lymph nodes and an abnormally enhanced lesion in the T9 vertebra, strongly indicating the possibility of distant metastasis. Based on the TNM staging system, we clinically classified this patient as T2N1M1 (stage IV). The patient’s largest tumor measured approximately 9.8 cm in diameter, and surgical resection would not be able to achieve both sufficient residual liver volume and negative surgical margins. For these reasons, our MDT initially deemed the tumor unresectable.

Conversion therapy refers to the reduction of tumor size and inhibition of tumor biological behavior through chemotherapy, radiotherapy, immunotherapy, targeted therapy, or combination therapies, thereby achieving radical resection and allowing patients to experience longer survival and an improved quality of life. With the advancement of systemic therapy for ICC, immunotherapy combined with targeted chemotherapy based on the GEMCIS regimen has shown promising results (3). The TOPAZ-1 trial proposed the combination of durvalumab with the GEMCIS regimen, demonstrating impressive efficacy in the systemic treatment of advanced biliary tract tumors, with a median survival time of 12.9 months, which was 50% higher than the placebo plus GEMCIS regimen (8). Similarly, the KEYNOTE-966 global phase III clinical trial evaluated the combination of pembrolizumab with the GEMCIS regimen and reported significantly improved median overall survival in the pembrolizumab group compared to the placebo group (12.7 months vs. 10.9 months) (9). Reports on the efficacy of targeted therapy combined with chemotherapy for advanced ICC remain limited. In a recently published single-center phase II clinical trial, the combination of toripalimab with lenvatinib and the GEMOX regimen achieved a median survival of up to 22.5 months, showing great potential as a first-line treatment for advanced ICC (9).

Our MDT consists of specialists from hepatobiliary surgery, hepatology, oncology, clinical pharmacy, radiology, and pathology. During our initial discussion of this case, we determined the TNM staging based on the patient’s auxiliary examinations and reached a consensus that the tumor was unresectable. Regarding systemic treatment, during the discussion, the oncologists recommended standard chemotherapy with the GC regimen (gemcitabine and cisplatin). However, considering the relatively poor median overall survival associated with chemotherapy alone, and the promising results from a recent single-center phase II clinical trial reporting a median OS of up to 22.5 months with a combination treatment of toripalimab, lenvatinib, oxaliplatin, and gemcitabine, the clinical pharmacy team suggested adding targeted therapy and immunotherapy to the chemotherapy regimen. After discussing the treatment plan with the patient, the final treatment strategy was determined to be GEMOX chemotherapy (gemcitabine + oxaliplatin) combined with lenvatinib as targeted therapy and toripalimab as immunotherapy. No tumor recurrence or distant metastasis was observed during the follow-up period. The potential mechanisms underlying the anti-tumor activity of this combination therapy include the following: (1) chemotherapy may improve the effectiveness of immunotherapy by reducing the immunosuppressive effect of the tumor microenvironment, increasing cross-presentation of tumor antigens, and promoting immune cell infiltration into the tumor core (10–12); and (2) targeted therapy may exert various immunostimulatory effects, including facilitating T-cell transport to the tumor and reducing immunosuppressive cytokines and regulatory T cells (13–15). With systemic therapy advancements, particularly in immunotherapy and targeted therapy, there are increasing reports of unresectable ICC cases being converted to resectable status and subsequently achieving successful tumor resection.

ALPPS rapidly increases liver volume within 1–2 weeks by ligating the portal vein and transecting the liver parenchyma, while PVE requires 4–8 weeks for hypertrophy by embolizing the portal vein branches (16). PVE may result in insufficient regeneration, delaying surgery or necessitating additional intervention, whereas ALPPS allows direct assessment of liver growth, ensuring feasibility (17). ALPPS is preferable for complex cases with extensive tumor invasion, whereas PVE may be less effective in controlling tumor progression (18). Despite a higher risk of complications, ALPPS selects patients with good regeneration potential, reducing liver failure risk, while PVE may cause thrombus extension, liver dysfunction, or tumor progression, especially with main portal vein involvement (19). This case highlights the potential of a multimodal approach in downstaging advanced ICC to enable curative resection. While this report describes a single patient, similar cases may benefit from a combination of chemotherapy, targeted therapy, and immunotherapy as conversion therapy. This case underscores the importance of individualized treatment strategies and real-time reassessment in managing advanced ICC. Future studies and case series are needed to evaluate the generalizability of this approach and refine patient selection criteria for multimodal conversion therapy.

## Conclusion

This case report demonstrates that unresectable advanced ICC can be converted into resectable ICC through a combination of immunotherapy, targeted therapy, and chemotherapy. The problem of insufficient residual liver volume can be effectively addressed using ALPPS. This study highlights that the GEMOX regimen combined with lenvatinib and toripalimab represents a potentially feasible and safe conversion treatment strategy for advanced ICC patients. This case contributes to the growing evidence supporting the use of multimodal therapy for achieving resectability and improving long-term survival in patients with advanced ICC.

## Data Availability

The original contributions presented in the study are included in the article/supplementary material. Further inquiries can be directed to the corresponding author.
